# Electronic Transport Mechanisms Correlated to Structural Properties of a Reduced Graphene Oxide Sponge

**DOI:** 10.3390/nano11102503

**Published:** 2021-09-26

**Authors:** Nicola Pinto, Benjamin McNaughton, Marco Minicucci, Milorad V. Milošević, Andrea Perali

**Affiliations:** 1 School of Science and Technology, Physics Division, University of Camerino, 62032 Camerino, Italy; benjamin.mcnaughton@unicam.it (B.M.); marco.minicucci@unicam.it (M.M.); 2 Advanced Materials Metrology and Life Science Division, INRiM (Istituto Nazionale di Ricerca Metrologica), Strade delle Cacce 91, 10135 Torino, Italy; 3 Department of Physics, University of Antwerp, Groenenborgerlaan 171, B-2020 Antwerp, Belgium; milorad.milosevic@uantwerpen.be; 4 School of Pharmacy, Physics Unit, University of Camerino, 62032 Camerino, Italy; andrea.perali@unicam.it

**Keywords:** graphene sponge, Raman, transport properties

## Abstract

We report morpho-structural properties and charge conduction mechanisms of a foamy “graphene sponge”, having a density as low as ≈0.07 kg/m${}^{3}$ and a carbon to oxygen ratio C:O ≃ 13:1. The spongy texture analysed by scanning electron microscopy is made of irregularly-shaped millimetres-sized small flakes, containing small crystallites with a typical size of ≃16.3 nm. A defect density as high as ≃2.6 × 10${}^{11}$ cm${}^{-2}$ has been estimated by the Raman intensity of *D* and *G* peaks, dominating the spectrum from room temperature down to ≃153 K. Despite the high C:O ratio, the graphene sponge exhibits an insulating electrical behavior, with a raise of the resistance value at ≃6 K up to 5 orders of magnitude with respect to the room temperature value. A variable range hopping (VRH) conduction, with a strong 2D character, dominates the charge carriers transport, from 300 K down to 20 K. At *T* < 20 K, graphene sponge resistance tends to saturate, suggesting a temperature-independent quantum tunnelling. The 2D-VRH conduction originates from structural disorder and is consistent with hopping of charge carriers between $sp^{2}$ defects in the plane, where $sp^{3}$ clusters related to oxygen functional groups act as potential barriers.

## 1. Introduction

Graphene-based compounds, such as graphene sponge (GS), are monolithic three-dimensional (3D) graphene-like structures that are gaining interest for their use in a variety of applications such as absorbers of pollutant liquids (e.g., oil), due to their super hydrophobicity [1], or as biosensors, owing to their large pore volume fraction [2], bioinspiring the design of graphene based materials [3]. GS-based transparent conductive electrodes were used in GaN-based blue light emitting diodes, improving the operating voltage by 26% and the output power by 14% [4]. Enhancement of photoresponse from the light-controlled conductive switching of nanocomposites based on Cu${}_{2}$O deposited on reduced graphene oxide has been found experimentally [5]. Energy storage and conversion is another attractive application of GSs, employing their tunable conductive inter-connected network and porous 3D structure, as well as high electrochemical and mechanical stability, which is beneficial for the development of fuel cells [6,7,8], batteries [6,7,9], super capacitors [6,10,11] and solar cells [6,12,13]. One should also note the strong photo-thermo-acoustic effect, with the generation of sound waves by fast temperature changes induced in a GS by intensity-modulated light [14,15].

To date, graphene sponges have been synthesised by a variety of well-developed methods such as chemical vapour deposition (CVD), self-assembly of graphene-oxide sheets with different reduction methods and freeze drying [16], all showing advantages and drawbacks as, for instance, a large variability of pore sizes, from 2 nm to few micrometers, deeply affecting the mechanical strength of the material [17]. While material fabricated by the relatively expensive CVD technique, may result in a nearly defect-free structure [18], cheap methods of synthesis as self-assembly, may originate highly-defective GS [19]. For this reason, other methods for cheap and sustainable production of carbon nanomaterials are a highly sought commodity [20]. Even though the macroscopic structure of graphene sponges is always 3D, the 2D-graphene backbone remains partially preserved, while material exhibits diverse quality and electrical properties, these last deeply affecting device design and working. In particular, investigation of the temperature dependent electronic transport properties allows understanding of charge carrier conduction mechanisms active in the material and it is a valuable tool to modify the fabrication process, aiming at graphene sponges with improved performance. Therefore, in this work we have studied the electrical resistance and the current-voltage characteristics as a function of temperature of a very low density graphene sponge made of graphene oxide with a high C:O ratio, purchased from Hygraner srl (for details, see Section 2). The low density of ≈0.07 kg/m${}^{3}$ makes the investigated GS potentially unique with respect to applications. Its electrical properties have been correlated to its microscopic morphology, composition and structure, while mechanisms explaining the charge transport have been suggested.

## 2. Experimental

The graphene sponge investigated in this work has been purchased by Hygraner srl (www.hygraner.it (accessed on 8 May 2012); product code: HYf-SG VW). All the technical information about the fabrication process here reported, has been taken from the Italian patent [see, https://worldwide.espacenet.com (accessed on 8 May 2012); publication number: ITUB20161052 (A1)—2017-08-25] with the addition of further details provided by the company. The material is synthesised by a solvo-thermal process starting from organic precursors based mainly on carbons, oxygen and hydrogen. Precursors have undergone a solvo-thermal process for more than 3 days at 250 ${}^{{}^{\circ}}$C, under nitrogen inert atmosphere, resulting in the production of graphene precursors, whose oxygen content can be tuned through the use of a polyol, varying the molar ratio between a metal and the polyol. Next step of the fabrication process is the pyrolysis, that deprives graphene precursors of hydrogen and oxygen resulting in the formation of graphene or graphene oxide. The pyrolysis is carried out in an oven, at temperatures ranging from 500 ${}^{{}^{\circ}}$C to 700 ${}^{{}^{\circ}}$C and for duration ranging from 30 s to 5 min, suitable to prevent oxidation in excess of the synthesised graphene. Then, the product undergoes a neutralisation step and it is washed with distilled water to reach a neutral pH. Finally, it is dried under vacuum and moderately heated. The graphene sponges obtained by this technique have a density ranging from 0.06 to 0.08 kg/m${}^{3}$, about one order of magnitude lower than graphene sponges studied in the literature.

The morphology and structure of our GS samples have been investigated by using an optical microscope Olympus mod. BX-60 and a field-emission scanning electron microscope (SEM) Zeiss Sigma-300 equipped with a Bruker Quantax energy dispersive (EDX) detector in order to collect x-rays for the micro-analysis of the elemental composition. Analysed samples have been fixed on aluminum sample holder (stubs) by using self-adhesive carbon conductive tabs. Up to 500,000× magnification scale has been achieved, allowing resolution down to few nanometers.

Raman spectroscopy of the GS has been carried out by a micro-Raman apparatus (Horiba mod. iHR320) including a Czerny-Turner spectrometer. The laser source (λ = 532 nm) has been operated at ≲1 mW to avoid sample heating and damaging. The setup includes a second laser source (λ = 632 nm), two Olympus microscopes (mod. BX41 and BXFM-ILHS) and a Linkam THMS600 cell for measurements in the 80÷873 K temperature range. We decided to show Raman spectra up to 153 K that is the temperature limit for a reasonable thermal stability of the low-T device with respect to our long acquisition time. This allowed to obtain both a good signal intensity and an optimal signal to noise ratio. Raman spectra have been collected by using a 600 mm${}^{-1}$ diffraction grating and collected through an objective having a magnification of 50×. The data obtained from Raman spectroscopy have been processed using the software Fityk [21], removing the background noise. All spectra have been normalised to the amplitude of the D-peak, before to proceed to their deconvolution. According to the fitting approach described by Ferrari and Robertson [22] the best deconvolution of peaks has been found by using a Lorentzian function for all bands, except in the case of the *G*-band for which a Voigt function has been used.

**Figure 1:**
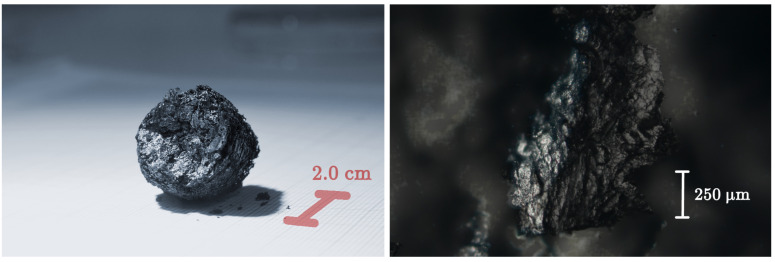
(**Left panel**): photo of the whole GS, investigated in this study, taken by a camera equipped with a macro lens. (**Right panel**): fragment of the GS observed by optical microscopy at a magnification of 5×. Eight images, each focussed at a different depth, have been combined together by a photo stacking technique to overcome the intrinsically 3D nature of the material and the reduced depth of field of the microscope.

For electrical investigation of GS properties, several fragments, having irregular shapes and different sizes, of the order of few millimetres, have been derived from a single macroscopic sample of GS (see Figure 1). Resistance and current-voltage (I-V) characteristics have been measured in the two contacts geometry, where two thin copper wires were fixed by two small droplets of silver print directly onto the surface of a single GS fragment. Special care has been paid to avoid mechanical strain and/or compression during contacts preparation and sample handling to avoid breaking of the fragment in the electrically contacted region, by means of a force in excess applied by the two copper wires. Silver print is proven effective to assure a good ohmic contact to GS fragments. A thin mylar film has assured electrical insulation of the sample from the cold finger, while optimal thermal contact with the cold finger has been guaranteed by a thin layer of Apiezon-N grease. All the electrical measurements have been carried out without applying any pressure to the samples.

I-V and R(T) characteristics have been measured as a function of temperature in a cryostat ARS mod. DE210 equipped with two Si diodes thermometers [23,24]. R(T) has been measured by an electrometer (Keithley mod. 6517B), operated in the V/I mode, applying a constant voltage bias (1 ÷ 10 V) and measuring the current, upon sample cooling down to about 6 K. Due to the high thermal inertia of the cryostat, data have been collected without any thermal stabilisation up to room temperature. The maximum temperature change has been ≲15 mK during the measurement of R(T) values to be averaged. At least 30 values have been averaged, for each point of the R(T) curve. The I-V characteristics, on the contrary, has been carried out after stabilising temperature within ±0.1 K, by using a Lakeshore mod. 332 controller. I-V data have been collected either by a source-meter Keysight mod. B2912A or by the electrometer.

## 3. Results

### 3.1. Structure

**Figure 2:**
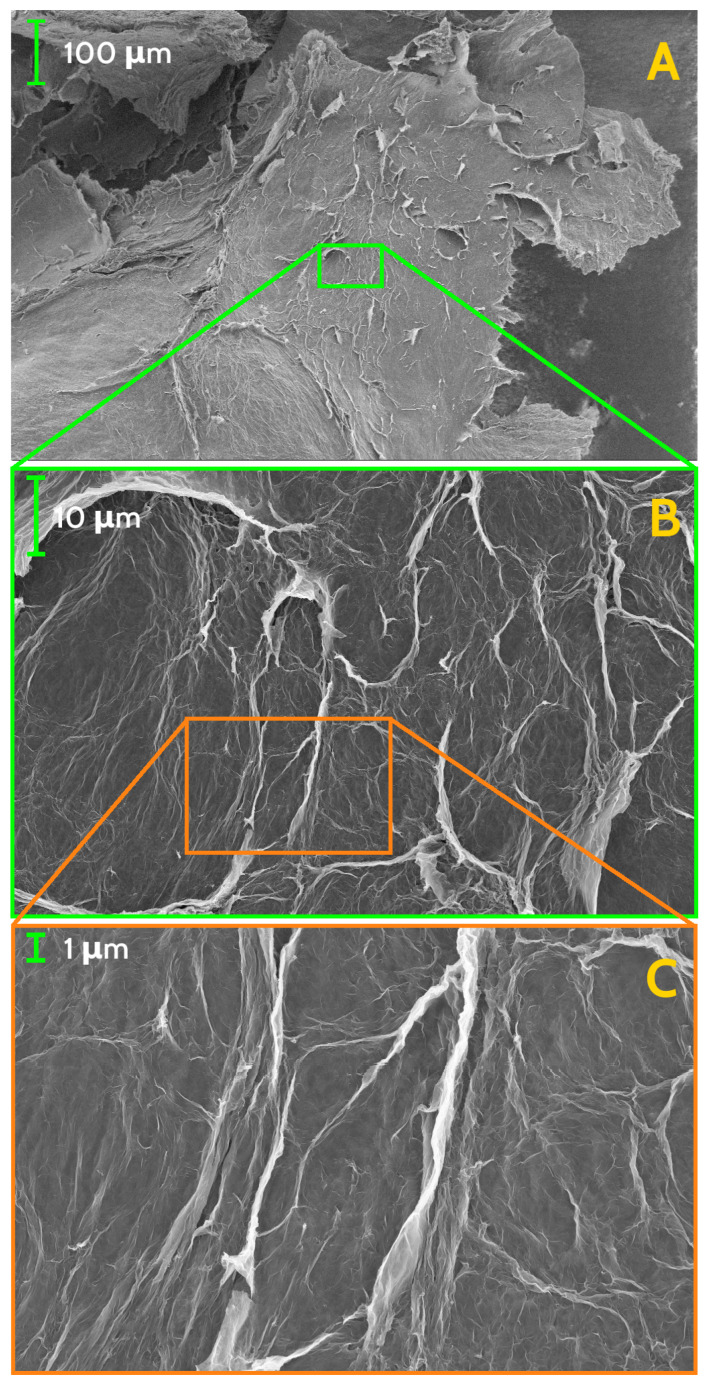
SEM images of the investigated GS, at different scale of magnification: (**A**) 244×, (**B**) 2000×, and (**C**) 10,000×. Long and compact lamellar structures are visible, closed in irregular geometries.

**Figure 3:**
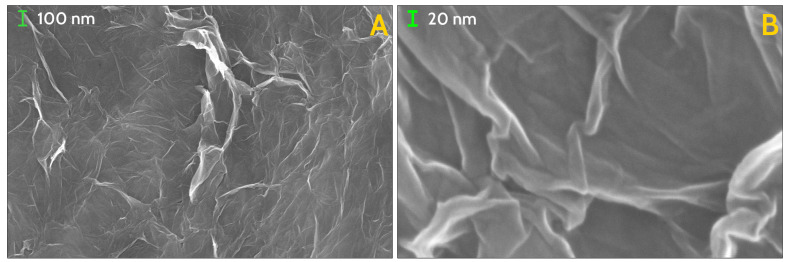
SEM images of the investigated GS, at scale of magnification of 100,000× (**A**) and 500,000× (**B**). Lamellar structures forming sharp angles are organised in closed paths at every scale of magnification, down to tens of nanometers.

Morphology of the GS has been investigated both by optical microscopy and by scanning electron microscopy (SEM), while homogeneity and local chemical composition has been probed by EDX analysis. On sight, the whole block of GS reveals continuous yet uneven surface, resembling a crumpled paper sheet (Figure 1, left panel), while optical microscopy, at low magnification, of a small fragment of the GS shows further features of the surface texture. Morphology details, gained by SEM at very low magnification, have evidenced wide and irregularly shaped flakes, with jagged borders, with typical size of few millimetres (Figure 2A). Flakes have a non-uniform thickness, appear to be stacked with a random orientation, leaving empty regions (i.e., cavities) in between up to few millimetres in size. At moderate magnification, a wrinkled surface of the flakes is visible, allowing to glimpse the spongy texture of the material (Figure 2B). The presence of irregular fine features is best seen at higher SEM magnification (Figure 2C and Figure 3A) while, at the highest achieved magnification, the uneven surface appears to be formed by an almost continuous and wrinkled quasi 2D layer, with thickness of ≈10÷20 nm (Figure 3B). Morphology and features of our GS are fairly similar to those observed by Stankovich et al. in their graphene sponge [19] and also to wrinkled few-layer graphene sheets seen in Ref. [25].

**Figure 4:**
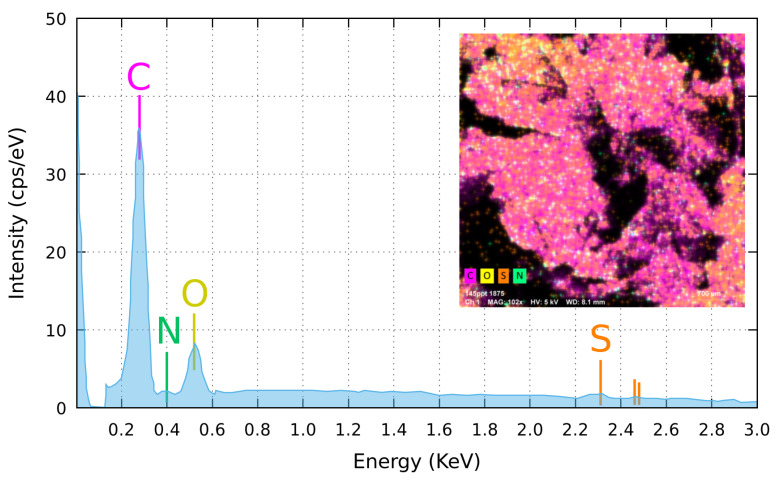
Averaged elemental composition of the GS, carried out by the EDX analysis on a sample surface 3.65×3.50 mm${}^{2}$ large. Inset: elemental composition map of the same area, evidencing a slightly patchy oxygen distribution, here appearing concentrated in the top-left and bottom-right corners of the analysed area. Visible black areas are due to holes in the sponge. Due to the lateral position of the EDX detector with respect to the sample (take-off angle of 35${}^{{}^{\circ}}$, at 8.5 mm above the specimen), signal cannot be collected from the black areas.

EDX micro-analysis, averaged on a millimetre sized area of a GS sample, has detected (see Table 1) carbon and oxygen as dominant presence, while nitrogen and sulphur gave only a negligible contribution to the measured signal that, for sulphur, is close to the detection limit of the EDX technique (Figure 4). Composition mapping has revealed a uniform distribution of carbon and a moderately uneven distribution of oxygen (Figure 4, inset). Data analysis showed an average C:O ratio of ≃13:1, close to that reported by Muzyka et al. for reduced graphene oxide [26] but higher than the value of ≈10:1 measured by Stankovich et al. for their reduced graphene oxide (rGO) [19]. The high C:O ratio detected in our GS classifies the material as highly reduced graphene oxide, hereafter mentioned as rGO.

### 3.2. Raman Spectrum

In what follows, we employ Raman spectroscopy to yield information on the quality of the GS material, average size of the crystallites, and the defect density. Information extracted from Raman spectra analysis will be correlated to features observed by SEM and to the electronic transport properties.

**Figure 5:**
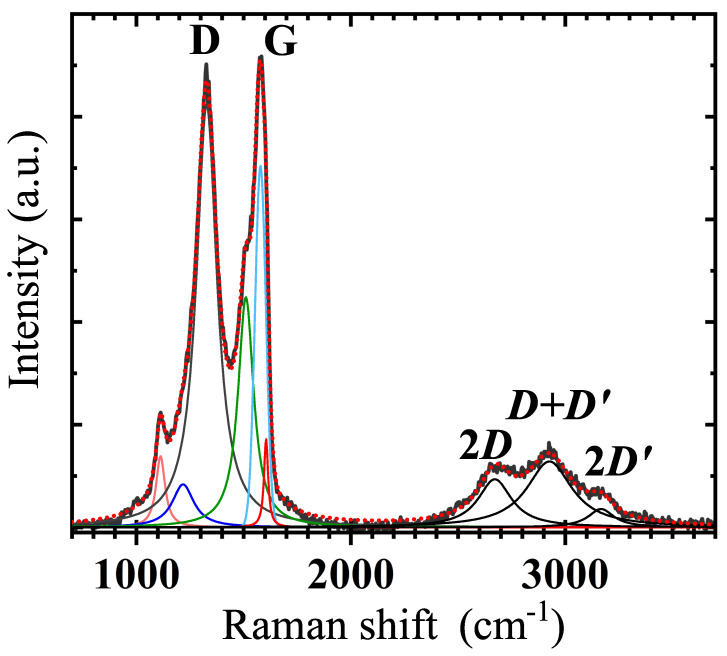
Raman spectrum of the GS at 293 K. The measured spectrum (thick and dark grey solid line) has been deconvoluted by Lorenzian and Voigt (*G*-peak only) functions into peaks and bands (coloured thin lines). Corresponding data are quoted in Table 2. The cumulative fitting curve is reproduced as red dots.

Raman spectrum of our GS at 293 K is shown in Figure 5, and evidences two prominent features, centred at 1578 cm${}^{-1}$ and at 1327 cm${}^{-1}$, conventionally labelled as *G*-peak and *D*-peak, respectively, next to a smaller peak seen at 1112 cm${}^{-1}$. Three broader and less intense second-order bands at 2671 cm${}^{-1}$, 2926 cm${}^{-1}$ and 3167 cm${}^{-1}$, are associated with two-dimensional (2D) carbon vibrational modes. Deconvolution of the *D* and *G*-peaks has revealed narrow components at 1217 cm${}^{-1}$, 1510 cm${}^{-1}$ and 1604 cm${}^{-1}$ (as shown in Figure 5). All results of the room temperature (RT) Raman spectrum analysis are quoted in Table 2.

The observed *G*-peak is the dominant signature for $sp^{2}$ electronic states (a first-order mode), having a linewidth of about 15 cm${}^{-1}$ for pure graphene, that usually undergoes broadening due to doping, temperature and strain [27]. In our GS the *G*-band appears blue-shifted with respect to the expected 1593 cm${}^{-1}$ (see Table 2 and references therein) due to the E${}_{2g}$ phonon mode of $sp^{2}$-bonded carbon atoms in a 2D hexagonal lattice [28]. In our GS, the overall profile of the *G*-peak is broadened (compared to 15 cm${}^{-1}$) possibly due to oxygen doping and strain caused by interconnected layers [25]. After deconvolution of the spectrum around the *G*-peak, we found two bands in the vicinity of the *G*-peak, denoted $D^{**}$ and $D^{\prime }$, that broaden the *G*-peak in the spectrum. An indicator of disorder and presence of defects in the material structure is the detection of the *D*-peak, whose amplitude is related to the defect density. As a result of a large variety of defects, the full width at half maximum (FWHM) of the *D*-peak can range from ≃5 cm${}^{-1}$ to hundreds of cm${}^{-1}$ [27]. The peak originates from the breathing modes of six carbon atom rings but requires defects for its activation. It stems from transverse-optical (TO) phonons around the K-edge of the Brillouin-zone and is active by a double-resonance [29]. In the investigated GS the *D*-peak exhibits a rather large FWHM (see Table 2), indicating presence of disorder, high density of defects and significant intervalley scattering [30,31,32].

**Table 2:** Peaks deconvolution of the Raman spectrum at 293 K shown in Figure 5. From left: conventional Raman peak label; expected peak position (cm${}^{-1}$), full width at half maximum (cm${}^{-1}$), peak position (cm${}^{-1}$) and relative peak amplitude (arbitrary units). The latter three quantities have been derived by peak analysis in this work. The expected peak positions are given and referenced with superscript labels a–i.

Raman Peak/Band	Position (Expected)	FWHM (meas.)	Position (meas.)	Amplitude (meas.)
	1100 ^a^	51	1112	0.1387
$D^{*}$	1200 ^b^	122	1217	0.0837
*D*	1327 ^c^	116	1327	0.8233
$D^{**}$	1500 ^d^	88	1510	0.4480
*G*	1593 ^e^	60	1578	0.7042
$D^{\prime }$	1610 ^f^	23	1604	0.1726
2D	2690 ^g^	190	2671	0.0937
$D+D^{\prime }$	2925 ^h^	267	2926	0.1284
$2D^{\prime }$	3160 ^i^	145	3167	0.0358

^a.^ [33], ^b.^ [25], ^c.^ [27], ^d.^ [25], ^e.^ [27], ^f.^ [25], ^g.^ [25], ^h.^ [25], ^i.^ [25].

On the left shoulder of the *D*-peak, we detected a peak at 1112 cm${}^{-1}$, that has been associated to $sp^{3}$-like bonds with eclipsed geometry, forming pentagonal rings in amorphous carbons [33]. Further, we find evidence of the $D^{*}$ and $D^{**}$ bands at 1217 cm${}^{-1}$ and 1510 cm${}^{-1}$, respectively, that can be associated with highly disordered and defective morphology of graphene sheets [25], correlating with the above discussed SEM analysis (see Figure 2). These bands have also been suggested to result from differences in disordered carbon, from C=C stretching and C-H wagging [25,34].

As mentioned before, the second-order region contains three bands, where the first occurring at 2671 cm${}^{-1}$ is referred to as the 2D band (Figure 5). At 2926 cm${}^{-1}$ we find the $D+D^{\prime }$ band (or D+G), and at 3167 cm${}^{-1}$ the $2D^{\prime }$ band [25]. The 2D band is the second-order mode of the *D* band, and can be used to quantify the number of layers of graphene in GS flakes, because the peak splits due to the evolution of electronic band structure [35,36]. The detection of a broad 2D band confirms our SEM observations about the presence of relatively large flakes of different thicknesses, as a result of a varying number of graphene-like layers from site to site (Figure 2a). The $D+D^{\prime }$ band originates from a combination of phonons with different momenta around *K* and Γ points of the reciprocal space, and requires the presence of defects for activation [36]. Finally, the $2D^{\prime }$ band is the second-order mode of the $D^{\prime }$ band at 1604 cm${}^{-1}$. The $D^{\prime }$ band originates from a double resonance via an intravalley process, by two connected points belonging to the same Dirac cone around *K* and $K^{\prime }$ [36].

### 3.3. Temperature Dependence

**Figure 6:**
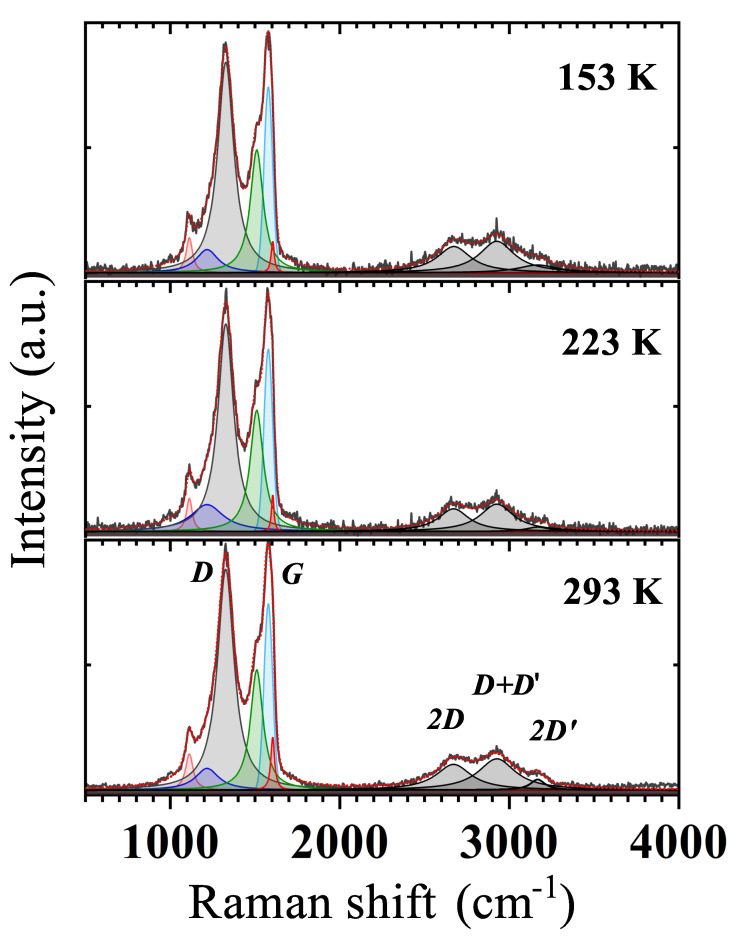
Raman spectra of the GS at different temperatures. Letters are used to label by convention the main peaks in the spectra. The Lorenzian and Voigt (*G*-peak only) peak contributions are shown as coloured shaded areas.

We have investigated the change of the previously detailed Raman peaks with temperature, collecting spectra below RT down to ≃153 K (Figure 6). Taken spectra show the maintained presence of all components detected at RT. However, a detailed analysis has revealed a weak *T* dependence of the *D* and *G*-peak intensity, used then to estimate the value of selected GS properties such as crystallites size $L_{a}$, the average distance between defects $L_{D}$, and the defect density $n_{def}$ [36,37]. Crystallites in our GS represent ordered graphitic regions surrounded by areas of oxidised carbon atoms or point defects [38] that, as shown later, are responsible for the mechanism of charge transport in this material.

From the *T*-dependence of the *D* to *G* peaks intensity ratio, $I_{D}/I_{G}$, and the laser wavelength, $\lambda_{L}=532$ nm, we have estimated $L_{a}$, $L_{D}$, and $n_{def}$ using the following empirical relations [36,37,39]:

(1) $$L_{a}=2.4\times 10^{-10}\lambda_{L}^{4}{\left(I_{D}/I_{G}\right)}^{-1},$$

(2) $$L_{D}^{2}=1.8\times 10^{-9}\lambda_{L}^{4}{\left(I_{D}/I_{G}\right)}^{-1},$$

and

(3) $$n_{def}=1.8\times 10^{22}\lambda_{L}^{-4}\left(I_{D}/I_{G}\right).$$

**Figure 7:**
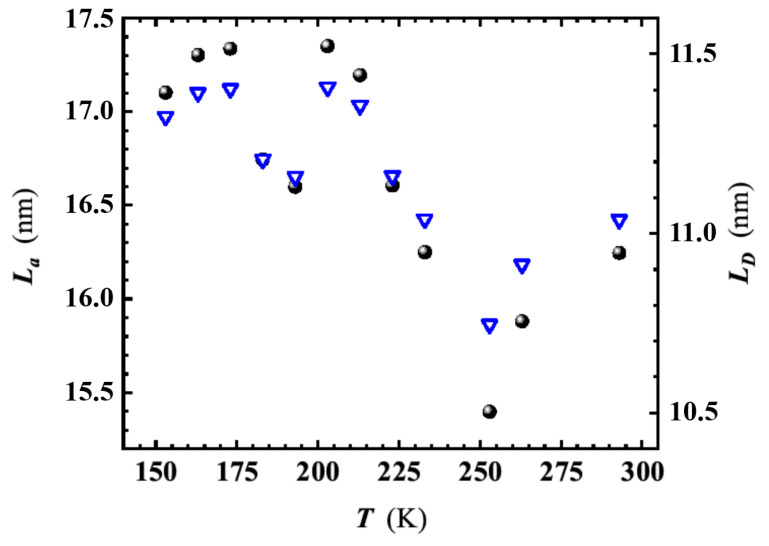
Temperature dependence of crystallites size, $L_{a}$, (dots) and distance between defects, $L_{D}$, (reversed triangles) in the investigated graphene sponge. $L_{a}$ and $L_{D}$ have been derived by using Equations (1) and (2), respectively. For the calculation, the Raman $I_{D}/I_{G}$ peak intensity ratio of the components at 1327 cm${}^{-1}$ (*D*-peak) and 1578 cm${}^{-1}$ (*G*-peak), respectively have been considered.

In the temperature range 153÷293 K, the $I_{D}/I_{G}$ ratio slightly decreases with lowering *T*, causing a moderate rise in the $L_{a}$ and $L_{D}$ values (as shown in Figure 7). On the other hand, the temperature dependence of $n_{def}$ appears negligible within the experimental error margin [36], resulting in an average value of $n_{def}≃2.6\times 10^{11}$ cm${}^{-2}$ in the considered temperature range. The $L_{D}$ behavior agrees with that found by Bhaskaram et al. for their rGO [39].

### 3.4. Transport Properties

**Figure 8:**
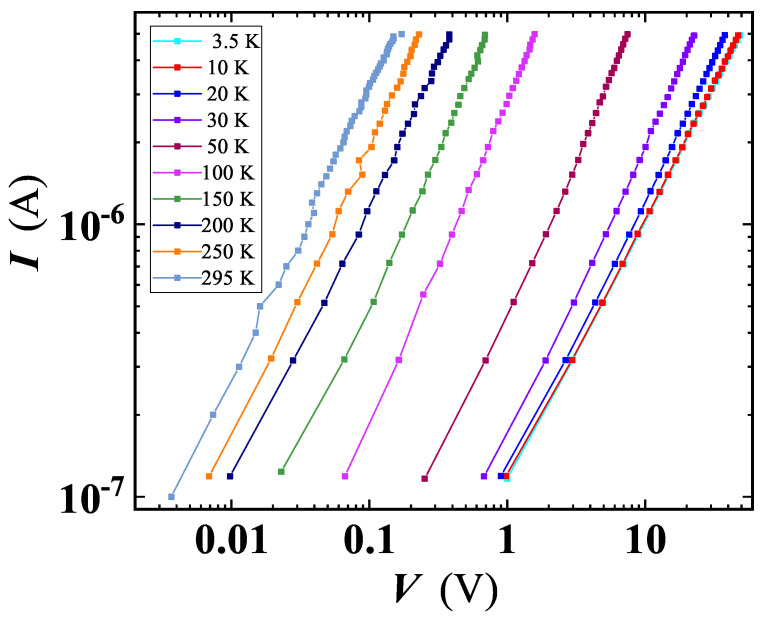
Current-voltage characteristics at several fixed temperatures, for a voltage-biased GS sample. Lines are shown as guides for the eyes.

To investigate electrical properties of our GS, we resorted to transport experiments on a voltage-biased sample. Current-voltage characteristics shown in Figure 8, taken at different temperatures, exhibit a linear (ohmic) behaviour with a negligible deviation from linearity at low applied voltages. This observed linearity claims that charge carrier injection from the metal contact interface is minimal and the conduction is bulk limited [40], while the drop of the current at fixed bias, with lowering *T*, points out the insulating character of the material.

**Figure 9:**
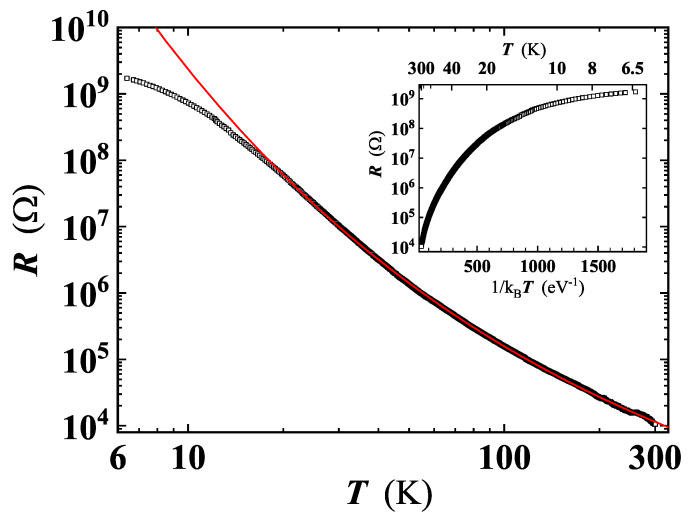
Resistance of the investigated GS as a function of temperature. Red line is the least-squares fit by Equation (4). Inset: Arrhenius plot of the same R(T) curve.

Details about carrier transport mechanisms have been inferred from the temperature dependence of the resistance, shown in Figure 9. Note that the particular texture and the morphology of the GS, also taking into account the absence of a defined geometry of the specimens, prevented us from converting the measured resistance into a resistivity value.

With decreasing temperature, we have found a rise up to 5 orders of magnitude in the resistance. In the sample shown in Figure 9, *R* increases from ≃$10^{4}$Ω at RT to ≃$2\times 10^{9}$Ω at ≃6 K, confirming the dominantly insulating character of the material. The Arrhenius plot of R(T) shows a monotonic decrease of the curve slope with lowering temperature (see the inset of Figure 9), excluding the thermally activated conduction as a mechanism for charge carriers transport in this system. The large variation of R(T), in the considered temperature range, can be explained by local non-uniformity of the C:O ratio, resulting in GO having different degree of reduction, in agreement with data of Joung and Khondaker, if the miminum *T* value of 6 K reached in our measurements is taken into account [41].

## 4. Discussion

Morphology and composition of graphene and graphene oxide based materials strongly influence their physical properties, and depend on the used fabrication technique. It is generally accepted that oxygen in the matrix introduces a high density of defected sites that, in turn, are expected to affect charge conduction mechanisms [39]. For instance, graphene oxide exhibits an insulating character [42,43] due to highly disordered regions covering a large fraction of the material, but parallel percolating conducting pathways develop between the electrodes upon removal of oxygen functional groups during the reduction process [44]. Hence, a close dependence between the electron transport properties of the GS and its microscopic morphology [45], as well as type and density of defects must be expected.

For graphene oxide and reduced graphene oxide, previous studies reported the observation of variable range hopping (VRH) conduction [39,41] usually occurring in a 2D system [38,44,46,47,48,49,50], with a temperature dependence of the resistance captured by the relation [41]:

(4) $$R\left(T\right)=R_{0}\exp\left[{\left(T_{0}/T\right)}^{1/(d+1)}\right],$$

where *d* is the effective dimensionality of the system in which charge carriers move, dependent on the density and degree of localisation of the states through which hopping transport of charge carriers occurs. $R_{0}$ is the pre-exponential factor and the expression for $T_{0}$ depends on the value of *d*.

**Figure 10:**
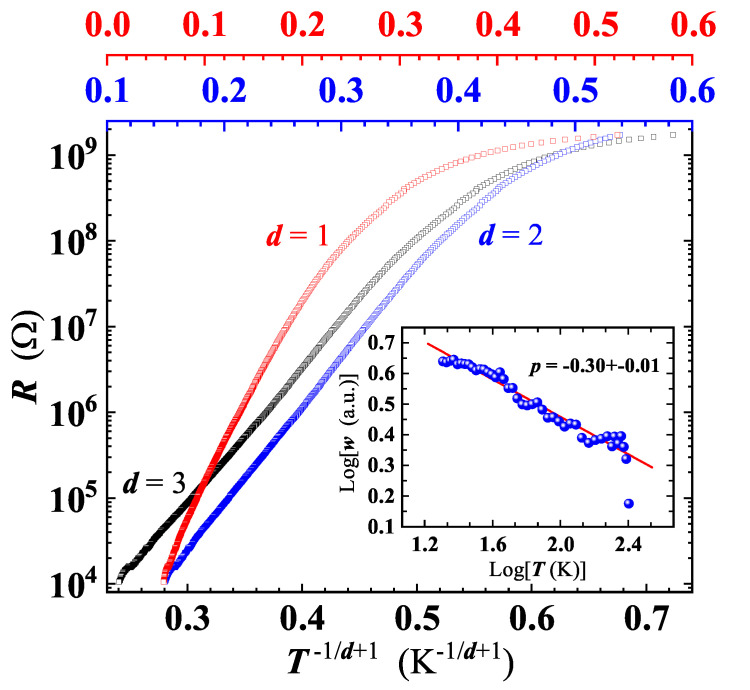
Analysis of the variable range hopping conduction mechanism in the GS, plotting the sample resistance as a function of: $T^{-1/4}$ (black squares, bottom axis); $T^{-1/3}$ (blue squares, intermediate-top axis) and $T^{-1/2}$ (red squares, top axis). Inset: reduced activation energy as a function of *T*, on a Log-Log scale. Plotted points are derived by applying the method described in the text to get the correct value of *d* in Equation (4). Red line is the linear least-squares fit of the plotted data, yielding the effective dimensionality d=2.3±0.11.

In our GS, VRH conduction has been checked by replotting R(T) data as a function of $T^{-1/(d+1)}$, with *d* = 1, 2 and 3 (Figure 10). While d=1 can be excluded, due to an evident non linearity of the curve, a difficulty arises in the choice between the other two *d* values.

The problem with identification of the correct *d* value is commonly faced in the analysis of the VRH conduction. In fact, the conventional plot of lnR(T) (or equivalently of lnρ(T)) as a function of $T^{-p}$ (here p=1/(d+1)) does not accurately determine *p*, as it implicitly assumes that the characteristics expressed by Equation (4) is obeyed [51]. A robust method to unambiguously identify the effective conduction mechanism involved (i.e., *p*), is to define a reduced activation energy, *w*, for R(T) as $w=T^{-1}\left(d\left[\ln R\left(T\right)\right]/dT^{-1}\right)$. Then, a reliable *p* value can be determined from the slope of logw vs. logT[41,51]. This approach has been proven valid to study the VRH conduction occurring in Ge and Si based nanostructures [51,52]. A least squares fit of our data in the log *w* vs. log *T* plot, yields p=0.30±0.01 (see inset of Figure 10) corresponding to d≃2.3, thus indicating a quasi-2D VRH transport mechanism, in agreement with the detection of high wavenumber broader bands in the Raman spectrum at all temperatures (Figure 6). The $T^{-1/3}$ dependence is fulfilled from RT down to ≈20 K, a significantly broader temperature range compared to those reported for GO and rGO [39,44]. A least squares fit of the R(T) curve by Equation (4) (Figure 9) has enabled us to obtain $T_{0}=\left(95,000\pm 5000\right)$ K, and to confirm that charge carriers transport indeed occurs in 2D, resulting with d=2.13±0.019, in good agreement with the value derived by the method described above. Below 20 K, the deviation of the R(T) behaviour from Equation (4) (Figure 9) suggests the existence of another conduction mechanism, as will be discussed later.

Having established that the VRH conduction occurs in 2D, it is now possible to use the appropriate expression for $T_{0}$ appearing in Equation (4) [40,47]:

(5) $$T_{0}=\frac{3\alpha^{2}}{k_{B}N\left(\epsilon_{F}\right)},$$

where $k_{B}$ and $N(\epsilon_{F})$ are the Boltzmann constant and the density of localised states (DOS) at the Fermi energy, respectively, while α is the wave function decay constant whose reciprocal value represents the charge carrier localisation length. An estimate of $N(\epsilon_{F})$ can be done assuming the size of crystallites $L_{a}$, taken from the Raman analysis, as a reasonable value for 1/α[40,53], considering also that $L_{a}$ has been previously found to be nearly independent on the level of reduction of GO [38,40]. $L_{a}$ must be considered as the upper limit for the localisation length of charge carriers, since smaller defects (e.g., point defects) may reduce indeed the 1/α value. Hence, assuming $1/\alpha =L_{a}≃16.3$ nm at RT (Figure 7) and the $T_{0}$ value found by the fit (Figure 9), using Equation (5) yields $N(\epsilon_{F})≃1.4$ × $10^{11}$ cm${}^{-2}$eV${}^{-1}$, comparable to the lower limit bound of the DOS values found by Eda et al. in GO with different level of reduction [40].

The density of states of GO and rGO has been studied theoretically and experimentally by several techniques [50,54,55]. In GO, DOS varies non-monotonically with energy, contains numerous gaps and peaks and its form has been predicted to depend on the oxygen coverage on the graphene surface. Upon GO reduction, the background DOS, due to graphene, re-emerges along with additional states due to defects [55]. Comparing to pristine graphene, the large number of structural defects in rGO can boost the graphene DOS at $\epsilon_{F}$, otherwise nearly zero in its pristine form [56]. The DOS involved in the VRH conduction of the charge carriers, will span a range of the order of ≈1 eV at $\epsilon_{F}$ [54,55] resulting in a number of states of the order of ≈$10^{11}$ cm${}^{-2}$, comparable to the defect density value calculated from the Raman data (Equation (3)).

On the basis of the above considerations, the detected VRH conduction is, at least in part, the electrical manifestation of the presence of defects and high disorder in the GS lattice matrix, as Raman investigation has clearly shown. However, for the assumptions above regarding 1/α, the value of $N(\epsilon_{F})$ represents, in any case, a lower limit, consistent with the possibility that additional physical causes, not producing visible features in the Raman spectrum, may contribute to the observed VRH conduction and to the increase of $N(\epsilon_{F})$.

For the further explanation of carrier conduction mechanisms occurring in the GS, we look deeper in the morphology of the investigated material. SEM observations and micro-analysis have revealed a morphology consisting of partially overlapped small flakes of rGO. These comprise conducting graphene-like $sp^{2}$ regions [57], as suggested by the presence of high wavenumber broader bands in the Raman spectrum (Figure 6) and by SEM analysis (Figure 3B). These $sp^{2}$ domains have a size of $L_{a}≃16.3$ nm, at RT, and are embedded in GO disordered regions [44,57], with a mean distance among defects of $L_{D}≃11$ nm at RT.

The insulating character of the rGO will depend on the fraction of oxidised disordered regions, forming clusters of $sp^{3}$ carbons in randomly distributed sizes, expected to give rise to a VRH conduction [49]. A strongly localised insulating state, promoting a 2D VRH conduction, has been found in graphene exposed to high doses of oxygen [48] as well as in lightly reduced GO [40]. In any case, the resistance values measured in our GS appear high for a reduced GO exhibiting a C:O ratio as high as 13:1, suggesting that they must originate from a highly defective structure, as captured by the Raman analysis.

**Figure 11:**
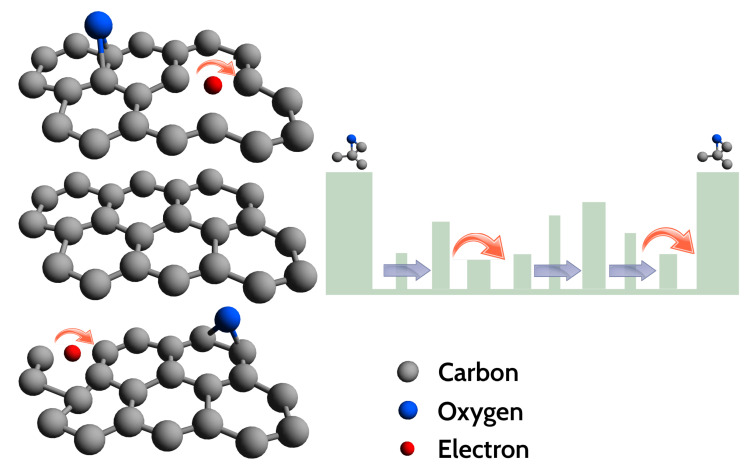
(**Left**): schematic illustration of the investigated graphene sponge structure, here assumed as formed by a build up of graphene-like planes, containing some defects and bridge bonded oxygen. (**Right**): VRH conduction of electrons trapped inside a large potential well created by the bonded oxygen. Charge carriers hop (red arrows) between two localised levels created by various defects present in the graphene plane. Blue arrows show a possible tunnelling mechanism occurring in the system.

As known in literature and mentioned above, a widespread defect is related to oxygen functional groups (with $sp^{3}$ hybridization), bridge-bonded to the graphene planes, supposed to create potential barriers between $sp^{2}$ hybridised crystalline and disordered regions [39,44,46,57]. It has been suggested before, that the barrier height depends on the local density of oxygen functional groups within the disordered regions and it can explain the large resistance that the VRH model predicts at low temperatures [44,46]. The high barriers at $sp^{3}$ clusters induce the formation of potential wells, trapping electrons [39,44]. A large network of $sp^{2}$-bonded carbon atoms, as well as additional defects inside these potential wells, will reduce the electron motion. On the contrary, $sp^{2}$ defects lying in the plane will have a lower barrier height as compared to the ones generated by the $sp^{3}$ clusters, so that charge carriers will prefer to hop across these defects, contributing to the VRH conduction detected in our experiments and in Refs. [44,46]. In other words, conduction of charge carriers will preferentially occur in 2D planes, in agreement with the $T^{-1/3}$ dependence of R(T) found by the analysis of the VRH conduction (Figure 9 and Figure 10). However, below ≈20 K, the R(T) curve progressively deviates from the $T^{-1/3}$ behaviour, exhibiting tendency to a saturation or to a resistance curve characterised by a lower slope. As known, at low *T*, the hopping conduction is ‘frozen’, explaining the deviation from the $T^{-1/3}$ dependence at T<20 K (Figure 9). Approaching the low-*T* range, a different conduction mechanism sets in, with a weak *T* dependence or completely *T*-independent. Considering the local structural features of our GS and the large defect density, the presence of small (thin) disordered regions, such as point and line defects, appears reasonable. Under these hypotheses, charge carriers can move throughout the material by quantum tunnelling, through thin barriers formed by the defects [44]. Quantum tunnelling, being independent on temperature, can explain the tendency to saturation of the R(T) curve. In a single layer of reduced graphene oxide, below about 25–40 K, Kaiser et al. [47] have found experimentally that the charge carriers transport is controlled by an electrically driven tunneling conduction mechanism, acting in parallel with a 2D-VRH, dominant at higher temperatures. However, in our GS, the I-V characteristics has not evidenced non-linearity effects that may suggest an applied electric field dependence of the conduction mechanisms. This fact can probably be explained by the different thickness of our samples and those investigated in the Ref. [47]. Based on the available resistance data the tunnelling mechanism remains a possible hypothesis, still to be confirmed via additional measurements extended to temperatures lower than 6 K, thus beyond the current limit of our measuring apparatus. A schematic drawing of the charge carriers transport mechanisms studied in our GS is depicted in Figure 11.

## 5. Conclusions

We here reported the morphology, composition, microscopic structure and charge conduction mechanisms of a low-density graphene sponge (commercially available from Hygraner srl). The studied material showed a visible foamy or spongy texture and an irregular morphology, formed by large and randomly oriented small flakes of graphene oxide, few millimetres in size. The composition analysis has proven carbon and oxygen as far dominant elements, in a C:O ratio close to 13:1, corresponding to that of a highly reduced graphene oxide. The Raman *D* and *G*-peaks, signature for $sp^{2}$ and $sp^{3}$ electronic states respectively, dominate the spectrum at all temperatures from RT down to about 153 K. From the temperature dependence of the intensity ratio of those peaks, we have estimated, at RT, a crystallites size of $L_{a}≃16.3$ nm, a distance among defects of $L_{D}≃11$ nm, and a defect density $n_{def}≃2.6\times 10^{11}$ cm${}^{-2}$ in the examined GS. All these quantities showed a minor or negligible dependence on temperature. In spite of the high level of reduction of GO, the GS exhibits an electrical insulating-like character, with resistance increasing up to 5 orders of magnitude when decreasing temperature from RT to about 6 K. The main conduction mechanism of charge carriers is the variable range hopping, detected in a broad temperature range, extending from RT down to ≈20 K. Our analysis has firmly established the effective two-dimensionality of the VRH, in agreement with the observation of high-wavenumber broader bands in the Raman spectrum, at all investigated temperatures. The electrical behaviour has then been related to the high density of defects and to disorder. Below 20 K, the tendency towards a saturation of the R(T) curve suggests a quantum tunnelling of charge carriers through thin barriers attributed to the possible presence of point and/or line defects.

To conclude, our experimental findings demonstrate a complex foamy and low density three dimensional graphene-like material, in which the interplay between the composition, the defected/irregular structure, and the random orientation of large flakes of graphene oxide, determine unconventional structural and electronic transport properties of interest for several technological applications.

## 6. Additional Information

Outcomes of the present study and the main conclusions here derived exclusively refer to the electrical behavior of the graphene sponge purchased from Hygraner srl. The sponge used for this study is produced for other uses (filtration). The material synthesis and the whole fabrication process reported in Section 2, has been derived from the patent document cited in this work and from additional information provided directly by Hygraner srl. The Authors have no direct or indirect interest in the Hygraner srl business.

## Figures and Tables

**Table 1:** Results of the EDX elemental composition analysis of the graphene sponge studied in this work. Here we show the element concentration in mass normalized [%] and atom [%] while in the 5th column the relative error at the one σ level is reported.

Element	Atomic No.	Mass Norm. [%]	Atom [%]	Rel. Error [%] (σ)
Carbon	6	89.72	92.15	11.15
Nitrogen	7	0.73	0.64	36.85
Oxygen	8	9.14	7.05	15.01
Sulfur	16	0.41	0.16	16.26

## Data Availability

The data that support the findings of this study are available from the corresponding author upon reasonable request.
